# Obstructive sleep apnoea increases lipolysis and deteriorates glucose homeostasis in patients with type 2 diabetes mellitus

**DOI:** 10.1038/s41598-021-83018-1

**Published:** 2021-02-11

**Authors:** Minh Duc Trinh, Andrea Plihalova, Jan Gojda, Katerina Westlake, Jan Spicka, Zuzana Lattova, Martin Pretl, Jan Polak

**Affiliations:** 1 Department of Pathophysiology, Third Faculty of Medicine, Charles University, Ruska 87, 100 00 Prague 10, Czech Republic; 2 Department of Cardiology, University Hospital Královské Vinohrady, Prague, Czech Republic; 3 Department of Internal Medicine, University Hospital Královské Vinohrady, Prague, Czech Republic; 4 Centre for Research on Diabetes, Metabolism and Nutrition, Third Faculty of Medicine, Charles University, Prague, Czech Republic; 5 Department of Laboratory Diagnostics, University Hospital Královské Vinohrady, Prague, Czech Republic; 6 Neurology and Sleep Laboratory, INSPAMED S.R.O., Prague, Czech Republic

**Keywords:** Endocrine system and metabolic diseases, Endocrine system and metabolic diseases

## Abstract

Obstructive sleep apnoea (OSA) is associated with type 2 diabetes mellitus (T2DM). However, mechanisms mediating association between these two conditions remain unclear. This study investigated, whether the OSA-associated changes in adipose tissue lipolysis might contribute to impaired glucose homeostasis in patient with T2DM. Thirty-five matched subjects were recruited into three groups: T2DM + severe OSA (T2DM + OSA, n = 11), T2DM with mild/no OSA (T2DM, n = 10) and healthy controls (n = 14). Subcutaneous abdominal adipose tissue microdialysis assessed spontaneous, epinephrine- and isoprenaline-stimulated lipolysis. Glucose metabolism was assessed by intravenous glucose tolerance test. Spontaneous lipolysis was higher in the T2DM + OSA compared with the T2DM (60.34 ± 23.40 vs. 42.53 ± 10.16 μmol/L, *p* = 0.013), as well as epinephrine-stimulated lipolysis (236.84 ± 103.90 vs. 167.39 ± 52.17 µmol/L, *p* < 0.001). Isoprenaline-stimulated lipolysis was unaffected by the presence of OSA (*p* = 0.750). The α_2_ anti-lipolytic effect was decreased in T2DM + OSA by 59% and 315% compared with T2DM and controls (*p* = 0.045 and *p* = 0.007, respectively). The severity of OSA (AHI) was positively associated with spontaneous (*p* = 0.037) and epinephrine-stimulated (*p* = 0.026) lipolysis. The α_2_-adrenergic anti-lipolytic effect (*p* = 0.043) decreased with increasing AHI. Spontaneous lipolysis was positively associated with Insulin resistance (r = 0.50, *p* = 0.002). Epinephrine-stimulated lipolysis was negatively associated with the Disposition index (r =  − 0.34, *p* = 0.048). AHI was positively associated with Insulin resistance (*p* = 0.017) and negatively with the Disposition index (*p* = 0.038). Severe OSA in patients with T2DM increased adipose tissue lipolysis, probably due to inhibition of the α_2_-adrenergic anti-lipolytic effect. We suggest that dysregulated lipolysis might contribute to OSA-associated impairments in insulin secretion and sensitivity.

## Introduction

Type 2 diabetes mellitus (T2DM) is one of the most prevalent medical conditions, affecting over 415 million people worldwide with estimated increase in prevalence by 2040 to 642 million adults^1^. Traditional risk factors for the development of T2DM, represented by obesity, age, physical inactivity and genetics have been well established; however, cross-sectional as well as prospective studies have demonstrated that obstructive sleep apnoea (OSA) is also associated with glucose intolerance, Insulin resistance and T2DM, independently of other risk factors^2–4^. OSA is a common disorder affecting 5% to 15% of middle–aged and older adults in the general population, but reaching up from 50 to 80% in T2DM or severely obese subjects^5^, characterized by episodic obstruction of the airway during sleep leading to repetitive oxyhaemoglobin desaturation and sleep fragmentation^6^.

Although the association between OSA and impaired glucose homeostasis was previously established^7–9^, mechanisms mediating this causal link remain unknown. The effect of intermittent hypoxia on glucose homeostasis has been proven in animal models as well as in healthy volunteers. Furthermore, exposure to intermittent hypoxia induced glucose intolerance, Insulin resistance, β-cell dysfunction and apoptosis, increased hepatic glucose output and stimulated adipose tissue lipolysis in mice^5,10–14^. Multiple intermittent-hypoxia induced metabolic derangements were only partially reversed after cessation of the exposure, whereas β-cell dysfunction gradually worsened^10^. Furthermore, similar effects were observed in humans, as acute exposure to intermittent hypoxia decreased insulin sensitivity and impaired β-cell function in healthy volunteers^15^. Among multiple suggested mechanisms (oxidative stress, increased sympathetic activity, elevated corticoid levels, elevated plasma endothelin-1 levels and activation of inflammatory pathways) increased levels of circulating free fatty acids (FFA) were recently proposed as the potential causal link between hypoxic exposure and impaired glucose metabolism^16,17^. Elevated circulating FFA levels impair glucose uptake and metabolism in muscle due to inhibition of key glycolytic enzymes^18,19^, induce Insulin resistance in liver, manifested as augmented hepatic glucose output, through effects on intracellular signalling pathways and gene expression^20–22^ and decrease insulin secretion and stimulate apoptosis in β-cells^23,24^. Furthermore, increased FFA levels further worsen metabolic control and insulin secretion in patients with T2DM^25,26^.

Importantly, adipose tissue oxygen levels as low as 4% O_2_ were observed during apnoeic episodes in a mouse model of OSA^27^, whereas in-vitro and rodent studies^5^ showed that lipolysis in adipocytes (a major source of circulating FFA) was up-regulated by low pericellular oxygen levels^28^. It is thus plausible to hypothesize that OSA-associated hypoxic episodes might augment adipose tissue lipolysis and stimulate release of FFA into circulation with its adverse metabolic consequences.

The aim of this study was to investigate whether adipose tissue lipolysis is modified by the presence of OSA in patients with T2DM to expand the knowledge on adipose-tissue pathophysiology in OSA, provide mechanistic links for epidemiological associations and identify possible treatment targets. To achieve this goal, we studied patients with T2DM with severe OSA compared to matched T2DM patients with mild/no OSA and control subjects without OSA. Subsequently, we assessed the impact of OSA on metabolic parameters such as Insulin resistance and β-cell function and we investigated the possible role of lipolysis—a proven drug target representing a novel treatment option for OSA-associated metabolic impairments^5^.

## Results

### Sleep, anthropometric and biochemical characteristics of recruited subjects

We enrolled 35 subjects (females n = 23 and males n = 12) in the study. The average age was 63.30 ± 5.75 years and BMI was 33.61 ± 3.24 kg/m^2^. Subjects diagnosed with T2DM and severe OSA presented with more apnoeic events reflected by AHI = 48.70 ± 17.43, compared with T2DM and healthy controls without OSA (AHI = 5.89 ± 2.90 and 4.32 ± 2.49, respectively, both *p* < 0.001). Likewise, T2DM subjects with severe OSA spent more time with haemoglobin saturation less than 90% (T90) compared with T2DM and healthy control groups (37.22 ± 32.07%, vs. 10.06 ± 12.85% and 1.88 ± 2.69%, respectively, *p* = 0.003 and *p* = 0.001, respectively).

There were no differences among groups in age and body composition (BMI, adiposity); however, waist circumference was higher in the T2DM + OSA group (111.91 ± 6.83 cm) compared with both the T2DM and control groups (104.50 ± 7.15 cm and 102.07 ± 8.39 cm, respectively, *p* = 0.033 and *p* = 0.003, respectively) as summarized in Table 1. Similarly, no differences were observed in plasma lipid, FFA and glycerol levels; nevertheless, subjects with severe OSA presented elevated plasmatic cortisol (by 20%), alanine transaminase (by 52%), and gamma-glutamyl transferase (by 224%) levels compared with T2DM group and the healthy controls, as shown in Table 2. Additionally, subjects diagnosed with T2DM showed 47% higher Insulin resistance (assessed by HOMA-IR) as well as 73% higher fasting plasma glucose and 69% higher HbA1c levels, independently of the presence/severity of OSA, as presented in Table 3. However, after adjustment the analysis for BMI, plasma insulin levels were higher in T2DM and T2DM + OSA group and similarly, after adjustment for gender, plasma insulin levels were higher in T2DM + OSA group compared to control group, as summarized in Supplementary material Tables A and B.

**Table 1:** Anthropometric and sleep characteristics of recruited subjects.

	Control	T2DM	T2DM + OSA
Subjects (n)	14	10	11
**Gender**			
Male	3 (21%)	3 (30%)	6 (55%)
Female	11 (79%)	7 (70%)	5 (45%)
Age (years)	62.18 (4.83)	63.95 (6.53)	64.13 (6.40)
Systolic blood pressure (mmHg)	133.86 (12.16)	137.30 (18.42)	141.36 (14.72)
Diastolic blood pressure (mmHg)	78.21 (9.97)	82.50 (11.95)	78.45 (24.76)
BMI (kg/m^2^)	33.50 (3.96)	32.72 (2.37)	34.56 (2.88)
Fat (kg)	29.56 (10.00)	27.59 (7.68)	28.69 (7.35)
Fat (%)	30.91 (9.55)	30.34 (8.51)	29.60 (8.49)
Waist circumference (cm)	102.07 (8.39)	104.50 (7.15)	111.91 (6.83)*†
AHI	4.32 (2.49)	5.89 (2.90)	48.70 (17.43)*†
ODI	4.29 (1.85)	6.80 (3.81)	46.23 (18.37)*†
T90 (%)	1.88 (2.69)	10.06 (12.85)	37.22 (32.07)*†
T85 (%)	0.18 (0.55)	0.37 (0.93)	10.96 (15.30)*†

Data are mean (SD), n (%).

BMI, body mass index; AHI = apnoea hypopnea index; ODI, oxygen desaturation index. T90 = percentage of total sleep time with oxygen saturation less than 90%. T85 = percentage of total sleep time with oxygen saturation less than 85%.

*Significant difference (*p* < 0.05) compared with control group.

^†^ Significant difference (*p* < 0.05) compared with T2DM group.

**Table 2:** Biochemical characteristics of recruited subjects.

	Control	T2DM	T2DM + OSA
**Total cholesterol (mmol/L)**	5.24 (0.64)	4.64 (1.15)	4.59 (0.77)
HDL (mmol/L)	1.52 (0.43)	1.22 (0.38)	1.30 (0.55)
LDL (mmol/L)	3.11 (0.69)	2.62 (1.09)	2.40 (0.83)
Triglycerides (mmol/L)	1.35 (0.57)	1.82 (0.75)	2.20 (1.52)*
FFA (mmol/L)	0.53 (0.14)	0.50 (0.11)	0.55 (0.16)
Glycerol (mmol/L)	137.86 (25.28)	139.27 (35.59)	145.75 (42.97)
Cortisol (nmol/L)	373.64 (72.88)	371.99 (91.39)	465.84 (135.63)*†
ALT (µkat/L)	0.39 (0.13)	0.45 (0.15)	0.63 (0.22)*†
AST (µkat/L)	0.43 (0.07)	0.37 (0.08)	0.45 (0.09)†
ALP (µkat/L)	1.13 (0.25)	1.24 (0.35)	1.20 (0.46)
GGT (µkat/L)	0.40 (0.15)	0.44 (0.26)	1.00 (1.07)*†

Data are mean (SD).

HDL, high density lipoprotein; LDL, low density lipoprotein; FFA, free fatty acids; ALT, alanine transaminase; AST, aspartate transaminase; ALP, alkaline phosphatase; GGT, gamma-glutamyl transferase.

*Significant difference (*p* < 0.05) compared with control group.

^†^ Significant difference (*p* < 0.05) compared with T2DM group.

**Table 3:** IVGTT results.

	Non-adjusted data			Adjusted data for waist circumference		
	Control	T2DM	T2DM + OSA	Control	T2DM	T2DM + OSA
Glucose (mmol/L)	5.54 (0.42)	7.68 (1.79)*	7.48 (1.58)*	5.39 (1.36)	7.63 (1.30)*	7.74 (1.44)*
HbA1c (mmol/mol)	36.21 (3.70)	52.70 (11.19)*	51.73 (12.47)*	35.71 (10.05)	52.52 (9.59)*	52.54 (10.54)*
Insulin (mU/L)	10.27 (4.00)	14.40 (4.30)	17.63 (8.90)*	11.67 (5.48)	14.88 (5.24)	15.20 (5.77)
HOMA-IR	2.55 (1.06)	4.86 (1.80)*	5.94 (3.50)*	2.85 (2.29)	4.96 (2.19)*	5.42 (2.41)*
AIR_g_ (mU L^−1 ^min^−1^)	708.57 (458.12)	371.60 (590.20)	151.97 (170.02)*	771.81 (452.62)	392.92 (432.90)*	42.11 (476.26)*
DI	1077.08 (642.85)	355.24 (576.91)*	174.16 (193.52)*	1090.98 (564.67)	359.93 (540.07)*	150.01 (594.15)*
S_I_ (mU/L^−1 ^min^−1^)	1.69 (0.88)	1.58 (1.26)	1.21 (0.45)	1.52 (0.91)	1.52 (0.87)	1.50 (0.96)
S_G_ (min^−1^)	0.02 (0.01)	0.01 (0.01)	0.02 (0.01)	0.02 (0.01)	0.01 (0.01)	0.01 (0.01)
β-Cell function (mU/mM)	125.30 (45.31)	109.23 (90.55)	119.99 (81.48)	142.92 (65.76)	115.17 (62.90)	89.38 (69.19)
Insulin resistance (mM mU L^−2^)	2.36 (0.95)	4.45 (1.42)*	5.47 (3.16)*	2.63 (2.01)	4.54 (1.92) *	5.00 (2.11)*

Data are mean (SD). HbA1c = glycated haemoglobin. HOMA-IR = homeostatic model assessment for Insulin resistance. AIR_g_—acute insulin response to glucose. DI—disposition index. S_I_ = insulin sensitivity. S_G_ = glucose effectiveness.

*Significant difference (*p* < 0.05) compared with control group.

Subjects with T2DM showed decreased Disposition index (reflecting the ability of pancreas to secrete insulin accordingly to prevalent Insulin resistance) by 23% and 201% increased Insulin resistance compared with healthy control subjects. Glucose-induced insulin secretion (represented by AIR_g_) was reduced by 35% in T2DM (not significantly) and further reduced by 79% (*p* < 0.05) in T2DM + OSA compared to a control group. After adjustment for waist circumference, reduction in AIR_g_ of T2DM and T2DM + OSA reached 49% and 95%, respectively (both *p* < 0.05). Controlling for gender, BMI or gender + BMI + waist circumference had no impact on AIR_g_ differences between groups, as summarized in Supplementary material Table C.

### Spontaneous, epinephrine, isoprenaline-stimulated lipolysis and α_2_-adrenergic anti-lipolytic effect

**Figure 1:**
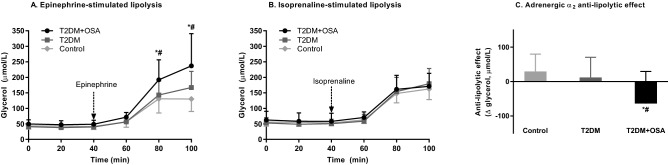
Lipolytic rate in subcutaneous abdominal adipose tissue. (**A**) Lipolytic rate after 10 μmol/L epinephrine administration in subcutaneous abdominal adipose tissue in all experimental groups. (**B**) Lipolytic rate after 10 μmol/L isoprenaline administration in subcutaneous abdominal adipose tissue in all experimental groups. (**C**) Presentation of the α_2_-anti-lipolytic effect in subcutaneous abdominal adipose tissue in all experimental groups. T2DM + OSA (n = 11), T2DM (n = 10), control group (n = 14). *p < 0.05 for differences between T2DM + OSA and a control group (repeated measures ANOVA with post-hoc tests). ^#^*p* < 0.05 for differences between T2DM + OSA and T2DM group (repeated measures ANOVA with post-hoc tests).

The investigation of the spontaneous lipolysis showed that the presence of severe OSA increased the lipolytic rate by 42% in T2DM + OSA compared with T2DM (60.34 ± 23.40 μmol/L vs. 42.53 ± 10.16 μmol/L, *p* = 0.013). Furthermore, the epinephrine-stimulated lipolysis was increased in T2DM + OSA by 41% and 82% compared with T2DM and healthy control subjects, respectively (236.84 ± 103.90 µmol/L vs. 167.39 ± 52.17 µmol/L and 130.33 ± 40.31 μmol/L, respectively, both *p* < 0.001). In contrast, no differences were observed after in-situ administration of isoprenaline (173.12 ± 43.40 μmol/L vs. 179.65 ± 49.19 μmol/L, for T2DM + OSA and T2DM, respectively, *p* = 0.750). Moreover, we investigated the α_2_-adrenergic anti-lipolytic effect in subcutaneous abdominal adipose tissue by evaluation of the difference between epinephrine-stimulated lipolysis (α- and β-adrenergic receptor agonist) and isoprenaline-stimulated lipolysis (selective β-adrenergic receptor agonist). The obtained data showed that the presence of severe OSA led to a decrease in the α_2_-adrenergic anti-lipolytic effect by 59% and 315% compared with the T2DM and control groups, respectively (*p* = 0.045 and *p* = 0.007, respectively). The data are summarized in Fig. 1.

### Correlations between lipolytic, metabolic and sleep parameters

Analysing the acquired results, we observed that AHI was positively associated with spontaneous lipolysis (*p* = 0.037) as well as epinephrine-stimulated lipolysis (*p* = 0.026). The severity of OSA (= AHI) was also associated with a diminished α_2_-adrenergic anti-lipolytic effect (*p* = 0.043). Moreover, spontaneous lipolysis was positively associated with Insulin resistance parameter derived from IVGTT (r = 0.50, *p* = 0.002), fasting plasma insulin levels (r = 0.40, *p* = 0.02) and HOMA-IR (r = 0.46, *p* = 0.006). Furthermore, epinephrine-stimulated lipolysis was negatively associated with the Disposition index (r =  − 0.34, *p* = 0.048).

AHI was positively associated with fasting plasma insulin (*p* = 0.033), an Insulin resistance parameter derived from IVGTT (*p* = 0.017) as well as measured by HOMA-IR (*p* = 0.018). Importantly, AHI was negatively associated with insulin secretion after intravenous glucose administration in the IVGTT (AUC_insulin_, *p* = 0.011) and the Disposition index (*p* = 0.038). Additionally, exposition to hypoxemia (= T90) was positively associated with Insulin resistance derived from IVGTT (r = 0.43, *p* = 0.012), HOMA-IR (r = 0.45, *p* = 0.008), fasting plasma insulin levels (r = 0.51, *p* = 0.002) and impaired insulin secretion after intravenous glucose administration (AUC_insulin_, r = 0.38, *p* = 0.026). As waist circumference showed a positive association with AHI (r = 0.60, *p* < 0.01), ODI (r = 0.59, *p* < 0.01) and T90 (r = 0.55, *p* = 0.001), we subsequently adjusted all IVGTT parameters for waist circumference, which had no impact on the outcomes.

## Discussion

In this study, we aimed to determine, whether the presence of severe OSA might contribute to impaired glucose homeostasis through augmented adipose tissue lipolysis in patients with already developed T2DM. Based on the fact that subcutaneous adipose tissue is the major contributor of FFA into the systemic pool^29^, an in-vivo in-situ microdialysis technique was employed together with metabolic phenotyping by IVGTT. We showed that the presence of severe OSA in subjects with T2DM increased spontaneous and epinephrine-stimulated lipolysis compared with T2DM subjects with mild/no OSA, possibly through modifications in the α_2_-adrenergic control of lipolysis. Furthermore, augmented adipose tissue lipolysis was associated with Insulin resistance and impaired insulin secretion, two clinical features determining the development and progression of T2DM.

The role of adipose tissue as a potential factor mediating connection between OSA and development of T2DM gained interest due to its proved impact on whole-body glucose metabolism^30,31^ as well as due to reports that adipocyte lipolysis is regulated by pericellular oxygen levels^5,16,28,32^. Previously, we demonstrated that adipocytes exposed to intermittent hypoxia in vitro (modelling oxygen desaturation in the context of OSA) increase spontaneous lipolytic rate^33^ which is in line with a current paper demonstrating higher FFA levels during IVGTT in subjects with OSA^34^ pointing to adipose tissue Insulin resistance. However, it remained unclear, whether such impairments persist in T2DM subjects with OSA as insulin resistance is present in these subjects. It could also be assumed that contribution of low O_2_ levels to lipolysis regulation is diminished in T2DM + OSA. We hypothesized that an OSA-associated increase in the release of FFA might not only induce an adipose tissue proinflammatory state^35^ and modify its endocrine function^36^, but also might impair insulin signalling through ectopic accumulation of lipids in muscle, liver and pancreas^37–39^. We observed that epinephrine-stimulated lipolysis, representing a model for nocturnal catecholamine bursts during apnoeic episodes, was elevated in T2DM patients with severe OSA, demonstrating that adipose tissue remained sensitive to β-adrenergic lipolytic drive even after prolonged exposure to periods of haemoglobin desaturations. Our study is in line with previous reports demonstrating that nocturnal hypoxemia elevated plasma FFA levels in heart failure subjects as well as after withdrawal from CPAP therapy^17,40^ and provides a mechanistic explanation of elevated plasma FFA levels. Using microdialysis technique, our study documented a link between adipose tissue hypoxia and spontaneous as well as epinephrine-stimulated lipolysis. Furthermore, results of our lipolysis experiments performed during awake hours suggest that increased adipose tissue lipolysis is not only the result of acute tissue hypoxemia during hypoxic episodes^16,40^, but it persists into wakefulness, probably due to prolonged effects on lipolysis regulation or due to persistently increased sympathetic nerve activity^41–44^. Indeed, exposure to hypoxia induces molecular changes in key lipolytic regulators^45^ and upregulation of its activators^46^, as well as by increased Insulin resistance to its anti-lipolytic effect^47^. On the other hand, exposure to hypoxia was reported to decrease FFA uptake and oxidation in muscle^48^, which would also result in FFA accumulation in the circulation.

The IVGTT, assessing simultaneously insulin sensitivity and glucose-induced insulin secretion, showed that AHI was associated with insulin secretion and Insulin resistance, supporting the previously reported association between OSA and T2DM^4^. Furthermore, epinephrine-stimulated lipolysis was negatively associated with the Disposition index—a variable describing the ability of β-cells to secrete insulin taking into account individual insulin sensitivity. Additionally, a link between enhanced adipose tissue lipolysis and development of Insulin resistance was reported previously^47,49^ and replicated in this study in subjects with T2DM and OSA, as spontaneous lipolysis was related to the severity of OSA and associated with multiple parameters of Insulin resistance. Augmented lipolysis during apnoeic episodes thus not only contributes to acute elevation of glucose levels^17^, but it also impairs insulin secretion and thus might participate in glycaemic control worsening in T2DM subjects or participate in the progressive pancreatic endocrine dysfunction. Although elevated FFA might represent an important mechanism inducing Insulin resistance and β-cell dysfunction, it should be noted that impaired insulin secretion, β-cell apoptosis, stimulated lipolysis and impaired muscle FFA metabolism were also observed in vitro as a direct consequence of hypoxia^28,50,51^. This study suggests a direct link between deoxygenation and metabolic impairments, as the level of hypoxic exposure (T90) was adversely associated with insulin sensitivity and insulin secretion.

Augmented sympathetic activity, as repeatedly reported in OSA^41,52,53^, was per se associated with metabolic impairments^54^; however, catecholamines also play a key role in the regulation of lipolysis through their binding to β- and α_2_-adrenergic receptors^55,56^. Studies showed that activation of β-adrenergic receptors stimulates lipolysis, while α_2_-adrenergic receptor activation has anti-lipolytic effect^57,58^. Administering epinephrine and isoprenaline into adipose tissue enabled assessment of individual contributions of α_2_ and β receptors to increased lipolysis. No differences in isoprenaline-stimulated lipolysis across groups suggest that hypoxia did not alter β-adrenergic receptor activation/signalling; however, lipolysis was significantly upregulated by epinephrine in OSA. A plausible explanation is that increased lipolysis observed in T2DM patients with OSA is mediated through diminished α_2_-adrenergic signalling. Indeed, the observation that pure β-adrenergic lipolysis is unaffected by hypoxia was reported by other authors as well. For example, no differences in isoprenaline-stimulated lipolysis were observed in normoxic vs intermittently hypoxic human isolated primary adipocytes^59^. Furthermore, modelling hypoxic responses by targeting HIF-signalling showed even reduced isoprenaline-stimulated lipolysis in human adipocytes ex vivo^60^ and exposing humans to hypoxic environment decreased isoprenaline-stimulated lipolysis^61^.

Interestingly, the severity of OSA was associated with the loss of α_2_ anti-lipolytic effect. Although verification of these observations is warranted, the fact that α_2_-adrenergic receptors outnumber β-adrenergic receptors in human adipose tissue^55^ makes α_2_-receptors not only a possible explanation for increased lipolysis in OSA, but also a potential pharmacological target for OSA-associated metabolic abnormalities, as suggested in a recent rodent study^5^.

Limitations of the study should be noted. First, experiments were performed in awake individuals, whereas apnoeic episodes occur during sleep, which could affect metabolite levels (e.g. plasma FFA), which remained unchanged in our study in contrast to studies using blood collections during sleep^17^. On the other hand, we were still able to detect changes in adipose tissue lipolysis persisting throughout the day, suggesting prolonged modifications in lipolysis regulation. Second, IVGTT does not assess the incretin-mediated part of insulin secretion (representing almost 65% of postprandial insulin secretion)^62^. Third, the limited sample size could affect statistical power of the present study, reflected in higher probability of type II error. For example, the power of isoprenaline-stimulated lipolysis comparison was 0.17, while for AIR_g_ and S_I_ the power was 0.38 and 0.37 respectively. Furthermore, unequal representation of the sexes warrants caution when generalizing results reported in the study as gender differences were reported in lipolysis regulation, response to weight reduction as well as other features associated with OSA^63,64^. The origin of referral/selection bias might be attributed to multiple sources, e.g. higher prevalence of diabetic women than men in the Czech Republic by 11% (according to the Institute of Health Information and Statistics of the Czech Republic), higher willingness of women to participate in a research study and recruitment through advertisement in local media with uneven gender distribution of readers. Importantly, there were no differences in basal or catecholamine–induced lipolysis between men and women in neither of the investigated groups, as summarized in Supplementary material Table D.

Finally, adipose tissue gene expression was not investigated in this study; however, previous studies reported no changes in α- or β-adrenergic receptor gene expression in visceral adipose tissue of OSA subjects compared with controls^65^.

In conclusion, the present study showed that the presence of OSA increased spontaneous lipolysis in T2DM patients, which was associated with the severity of OSA, as well as with Insulin resistance and impaired insulin secretion. The study identified reduced α_2_ anti-lipolytic effect as a possible factor responsible for increased lipolysis in severe OSA. Based on these observations, we suggest that pharmacological inhibitors of lipolysis might represent a novel treatment modality for metabolic impairments associated with OSA in T2DM, particularly for subjects not tolerating CPAP therapy. Moreover, despite the strong evidence of relationship between OSA and T2DM, studies investigating the effect of CPAP treatment on glycaemic control in T2DM provide inconclusive results (with multiple differences in study designs). For example, no effect of CPAP treatment was observed in GlycOSA study^66^, while another study^67^ showed beneficial effects of CPAP on glucose control similarly to the largest and longest running clinical trial to date, the SAVE substudy, which reported benefits of CPAP therapy, although limited to women^68^.

## Methods

### Subjects

The subjects were recruited through referral from physicians and local media advertisement. Subjects were recruited into three groups: patients with T2DM with mild/no OSA (T2DM, n = 10, 7 postmenopausal females), patients with T2DM with severe OSA (T2DM + OSA, n = 11, 5 postmenopausal females) and healthy controls without OSA or T2DM (n = 14, 11 postmenopausal females). The inclusion criteria were set as 18 to 85 years of age and body mass index (BMI) of 22 to 40 kg/m^2^. T2DM was diagnosed based on the European Association for the Study of Diabetes criteria^69^. Importantly, all patients with acute illness, decompensated chronic disease, cardiac or renal insufficiency, treated with drugs interacting with lipolysis (i.e., beta blockers, corticoids, insulin treatment, sulfonylurea treatment, GLP-1 receptor agonists and gliflozins) and a body weight change > 5 kg in last 3 months were excluded. All subjects gave written informed consent before participation in the study. The study was registered in ClinicalTrials.gov (NCT02683616) on 17.02.2016 and approved by the Ethical Committee of the University Hospital Královské Vinohrady, Prague. All methods were carried out in accordance with relevant guidelines and regulations (Declaration of Helsinki).

### Sleep study

The sleep recordings were performed using a type III device recording haemoglobin saturation, heart rate, electrocardiogram, nasal airflow, chest and abdominal respiratory efforts (Nox T3, Nox Medical, Reykjavik, Iceland) in a home setting. The acquired data were evaluated by a board-certified sleep physician according to the American Academy of Sleep Medicine criteria (apnoea defined as a ≥ 90% reduction in airflow for at least 10 s and hypopnea defined as a ≥ 30% reduction in airflow for at least 10 s together with ≥ 4% desaturation). The severity of OSA was classified by AHI: < 5 no OSA, AHI ≥ 5 and < 15 mild OSA, AHI ≥ 15 and < 30 moderate OSA, AHI ≥ 30 severe OSA.

### Microdialysis

Microdialysis was performed at 8:00 AM after overnight fasting. Three 20 kDa microdialysis catheters (63 Microdialysis catheter, CMA Microdialysis AB, Kista, Sweden) were inserted into the subcutaneous abdominal adipose tissue after local epidermal anaesthesia (1% Mesocaine, Zentiva a.s., Prague, Czech Republic). Catheter number 1. was perfused with Ringer’s solution to assess spontaneous non-stimulated lipolysis. Catheter number 2. was perfused with 10 μmol/L epinephrine (α- and β-adrenergic receptor agonist) while catheter number 3. was perfused with 10 μmol/L isoprenaline (selective β-adrenergic receptor agonist). Each catheter was connected to a micro-perfusion pump (Harvard Apparatus, Holliston, Massachusetts, USA). The outflowing dialysate was collected every 20 min. During the initial equilibration period (120 min) when catheters were perfused with Ringer’s solution, the flow rate was gradually decreased to final flow rate of 2.5 µL/min, which was used throughout the experiment. Subsequently three basal samples were collected to determine spontaneous lipolytic rate (perfusion with Ringer’s solution only), and the perfusion fluid was changed to epinephrine for catheter number 2 and isoprenaline for catheter number 3. Catheter number 1 remained perfused with Ringer’s solution. Pharmacological perfusion lasted an additional 60 min. All dialysate samples were immediately frozen and stored at − 80 °C until analysis.

### Biochemical analysis

For purposes of lipolysis assessment, glycerol concentration in dialysate was determined and used as a marker of lipolysis (Free glycerol reagent F6428, Sigma-Aldrich, St. Louis, MI, USA). FFA in serum were measured by NEFA-HR2 assay (Wako Chemical Inc., Richmond, VA, USA). All the other biochemical analyses were performed by the institutional Department of Laboratory Diagnostics, Kralovske Vinohrady University Hospital, Prague.

### Clinical investigations

Within 2 weeks of the microdialysis experiment, subjects visited the research centre for metabolic and anthropometric assessments. The measurements included blood drawn for biochemical analysis, blood count, coagulation and urinalysis, multifrequency bioimpedance measurements (Body Impedance analyser NUTRIGUARD-M, Data Input GmbH, Frankfurt, Germany) for body composition data and recording of individual body weight, height and waist circumference. Subsequently, a frequent-sampling intravenous glucose tolerance test (IVGTT) was performed. as follows: two intravenous catheters were inserted in the antecubital vein in the dominant (for blood sampling) and non-dominant (for glucose and insulin administration) arm. Basal sampling at times − 15, − 10, − 5, and − 1 min were performed, followed by intravenous administration of 0.3 g/kg glucose at time 0 min. Twenty minutes after the glucose dose, 0.03 U/kg of insulin (Humulin R, Lilly France S.A.S, Fegersheim, France) was administered intravenously. Blood samples were collected at times 2, 3, 4, 5, 6, 8, 10, 12, 14, 16, 19, 22, 24, 25, 27, 30, 40, 50, 60, 70, 80, 90, 100, 120, 140, 160, and 180 min. The glucose and insulin levels in collected samples were determined. The values were subjected to the minimal model analysis^70^ for insulin sensitivity and insulin secretion indices, including acute insulin response to glucose (AIR_G_), Disposition index (DI), Insulin sensitivity (S_I_), Glucose effectiveness (S_G_), β-cell function, and Insulin resistance.

### Statistical analysis

The differences in outcome variables between groups (control, T2DM and T2DM + OSA) were analysed using one-way analysis of variance (ANOVA) with least significant difference post-hoc tests. Repeated measures ANOVA was employed to analyse differences between groups in epinephrine and isoprenaline-stimulated lipolysis. Correlations between continuous variables were analysed using the Pearson correlation coefficient. For the investigation of associations between outcome variables and severity of OSA, all subjects were first stratified into groups based on terciles of AHI and subsequently subjected to analysis using the general linear model adjusted for sex and age. For statistical tests, SPSS 23.0 for Windows (SPSS Inc., Chicago, IL, USA) was used, whereas GraphPad Prism 7 (GraphPad Software Inc., La Jolla, CA, USA) was used for AUC_insulin_ calculation and figure construction. The statistical significance was set to, *p* ≤ 0.05 in all tests. Data are presented as mean ± standard deviation.

## Supplementary Information

Supplementary Information.

## Data Availability

The datasets generated during and/or analysed during the current study are available from the corresponding author on reasonable request.

## References

[1] Zheng Y, Ley SH, Hu FB (2017). Global aetiology and epidemiology of type 2 diabetes mellitus and its complications. Nat. Rev. Endocrinol..

[2] Briançon-Marjollet A (2015). The impact of sleep disorders on glucose metabolism: endocrine and molecular mechanisms. Diabetol. Metab. Syndr..

[3] Tasali E, Mokhlesi B, Van Cauter E (2008). Obstructive sleep apnea and type 2 diabetes. Chest.

[4] Aurora RN, Punjabi NM (2013). Obstructive sleep apnoea and type 2 diabetes mellitus: a bidirectional association. Lancet Respir. Med..

[5] Weiszenstein M, Shimoda LA, Koc M, Seda O, Polak J (2016). Inhibition of lipolysis ameliorates diabetic phenotype in a mouse model of obstructive sleep apnea. Am. J. Respir. Cell Mol. Biol..

[6] Neubauer JA (2001). Invited review: physiological and pathophysiological responses to intermittent hypoxia. J. Appl. Physiol..

[7] Steiropoulos P, Papanas N, Bouros D, Maltezos E (2010). Obstructive sleep apnea aggravates glycemic control across the continuum of glucose homeostasis. Am. J. Respir. Crit Care Med..

[8] Pugliese G (2020). Sleep apnea, obesity, and disturbed glucose homeostasis: epidemiologic evidence, biologic insights, and therapeutic strategies. Curr. Obesity Rep..

[9] Pallayova M (2010). Sleep apnea predicts distinct alterations in glucose homeostasis and biomarkers in obese adults with normal and impaired glucose metabolism. Cardiovasc. Diabetol..

[10] Polak J (2013). Intermittent hypoxia impairs glucose homeostasis in C57BL6/J mice: partial improvement with cessation of the exposure. Sleep.

[11] Polotsky VY (2003). Intermittent hypoxia increases insulin resistance in genetically obese mice. J. Physiol..

[12] Iiyori N (2007). Intermittent hypoxia causes insulin resistance in lean mice independent of autonomic activity. Am. J. Respir. Crit Care Med..

[13] Xu J, Long Y-S, Gozal D, Epstein PN (2009). Beta-cell death and proliferation after intermittent hypoxia: role of oxidative stress. Free Radic. Biol. Med..

[14] Yokoe T (2008). Intermittent hypoxia reverses the diurnal glucose rhythm and causes pancreatic β-cell replication in mice. J. Physiol..

[15] Louis M, Punjabi NM (2009). Effects of acute intermittent hypoxia on glucose metabolism in awake healthy volunteers. J. Appl. Physiol..

[16] Plihalova A (2016). The effect of hypoxia and re-oxygenation on adipose tissue lipolysis in COPD patients. Eur. Respir. J..

[17] Chopra S (2017). Obstructive sleep apnea dynamically increases nocturnal plasma free fatty acids, glucose, and cortisol during sleep. J. Clin. Endocrinol. Metab..

[18] Kelley DE, Goodpaster B, Wing RR, Simoneau JA (1999). Skeletal muscle fatty acid metabolism in association with insulin resistance, obesity, and weight loss. Am. J. Physiol..

[19] Kelley DE, Mandarino LJ (2000). Fuel selection in human skeletal muscle in insulin resistance: a reexamination. Diabetes.

[20] Boden G, Chen X, Capulong E, Mozzoli M (2001). Effects of free fatty acids on gluconeogenesis and autoregulation of glucose production in type 2 diabetes 1. Diabetes.

[21] Samuel VT, Petersen KF, Shulman GI (2010). Lipid-induced insulin resistance: unravelling the mechanism. Lancet.

[22] Pereira S (2014). FFA-induced hepatic insulin resistance in vivo is mediated by PKCδ, NADPH oxidase, and oxidative stress. Am. J. Physiol. Endocrinol. Metab..

[23] Cnop M (2005). Mechanisms of pancreatic beta-cell death in type 1 and type 2 diabetes: many differences, few similarities. Diabetes.

[24] Acosta-Montaño P, García-González V (2018). Effects of dietary fatty acids in pancreatic beta cell metabolism, implications in homeostasis. Nutrients.

[25] Aronsohn RS, Whitmore H, Van Cauter E, Tasali E (2010). Impact of untreated obstructive sleep apnea on glucose control in type 2 diabetes. Am. J. Respir. Crit. Care Med..

[26] Tahrani AA (2012). Obstructive sleep apnea and diabetic neuropathy. Am. J. Respir. Crit. Care Med..

[27] Reinke C, Bevans-Fonti S, Drager LF, Shin M-K, Polotsky VY (2011). Effects of different acute hypoxic regimens on tissue oxygen profiles and metabolic outcomes. J. Appl. Physiol..

[28] Weiszenstein M (2016). Adipogenesis, lipogenesis and lipolysis is stimulated by mild but not severe hypoxia in 3T3-L1 cells. Biochem. Biophys. Res. Commun..

[29] Jensen MD (2002). Adipose tissue and fatty acid metabolism in humans. J. R. Soc. Med..

[30] Hajer GR, van Haeften TW, Visseren FLJ (2008). Adipose tissue dysfunction in obesity, diabetes, and vascular diseases. Eur. Heart J..

[31] Winkler G (2003). Expression of tumor necrosis factor (TNF)-alpha protein in the subcutaneous and visceral adipose tissue in correlation with adipocyte cell volume, serum TNF-alpha, soluble serum TNF-receptor-2 concentrations and C-peptide level. Eur. J. Endocrinol..

[32] Ye J (2009). Emerging role of adipose tissue hypoxia in obesity and insulin resistance. Int. J. Obes. (Lond.).

[33] Musutova M, Weiszenstein M, Koc M, Polak J (2020). Intermittent hypoxia stimulates lipolysis, but inhibits differentiation and de novo lipogenesis in 3T3-L1 cells. Metab. Syndr. Relat. Disord..

[34] Stefanovski D, Boston RC, Punjabi NM (2020). Sleep-disordered breathing and free fatty acid metabolism. Chest.

[35] Boden G (2008). Obesity and free fatty acids. Endocrinol. Metab. Clin. North Am..

[36] Jung UJ, Choi M-S (2014). Obesity and its metabolic complications: the role of adipokines and the relationship between obesity, inflammation, insulin resistance, dyslipidemia and nonalcoholic fatty liver disease. Int. J. Mol. Sci..

[37] Frayn KN, Tan GD, Karpe F (2007). Adipose tissue: a key target for diabetes pathophysiology and treatment?. Horm. Metab. Res..

[38] Bergman RN, Ader M (2000). Free fatty acids and pathogenesis of type 2 diabetes mellitus 1. Trends Endocrinol. Metab..

[39] Boden G (2003). Effects of free fatty acids (ffa) on glucose metabolism: significance for insulin resistance and type 2 diabetes. Exp. Clin. Endocrinol. Diabetes.

[40] Jun JC (2011). Effects of sleep apnea on nocturnal free fatty acids in subjects with heart failure. Sleep.

[41] Narkiewicz K, Somers VK (2003). Sympathetic nerve activity in obstructive sleep apnoea. Acta Physiol. Scand..

[42] Prabhakar NR, Kumar GK (2010). Mechanisms of sympathetic activation and blood pressure elevation by intermittent hypoxia. Respir. Physiol Neurobiol..

[43] Spaak J (2005). Muscle sympathetic nerve activity during wakefulness in heart failure patients with and without sleep apnea. Hypertension.

[44] Kasai T, Bradley TD (2011). Obstructive sleep apnea and heart failure: pathophysiologic and therapeutic implications. J. Am. Coll. Cardiol..

[45] Miyoshi H, Perfield JW, Obin MS, Greenberg AS (2008). Adipose triglyceride lipase regulates basal lipolysis and lipid droplet size in adipocytes. J. Cell Biochem..

[46] Pagnon J (2012). Identification and functional characterization of protein kinase A phosphorylation sites in the major lipolytic protein, adipose triglyceride lipase. Endocrinology.

[47] Morigny P, Houssier M, Mouisel E, Langin D (2016). Adipocyte lipolysis and insulin resistance. Biochimie.

[48] Musutova M (2018). The effect of hypoxia and metformin on fatty acid uptake, storage, and oxidation in L6 differentiated myotubes. Front. Endocrinol. (Lausanne).

[49] Girousse A (2013). Partial inhibition of adipose tissue lipolysis improves glucose metabolism and insulin sensitivity without alteration of fat mass. PLoS Biol..

[50] Wang N, Khan SA, Prabhakar NR, Nanduri J (2013). Impairment of pancreatic beta-cell function by chronic intermittent hypoxia. Exp. Physiol.

[51] Weiszenstein M (2016). The effect of pericellular oxygen levels on proteomic profile and lipogenesis in 3T3-L1 differentiated preadipocytes cultured on gas-permeable cultureware. PLoS ONE.

[52] Bisogni V, Pengo MF, Maiolino G, Rossi GP (2016). The sympathetic nervous system and catecholamines metabolism in obstructive sleep apnoea. J. Thorac. Dis..

[53] Roder F (2018). Interactions of sleep apnea, the autonomic nervous system, and its impact on cardiac arrhythmias. Curr. Sleep Med. Rep..

[54] Thorp AA, Schlaich MP (2015). Relevance of sympathetic nervous system activation in obesity and metabolic syndrome. J. Diabetes Res..

[55] Stich V (2003). Activation of alpha2-adrenergic receptors blunts epinephrine-induced lipolysis in subcutaneous adipose tissue during a hyperinsulinemic euglycemic clamp in men 1. Am. J. Physiol Endocrinol. Metab..

[56] Collins S (2011). β-adrenoceptor signaling networks in adipocytes for recruiting stored fat and energy expenditure. Front. Endocrinol. (Lausanne).

[57] Lafontan M, Berlan M (1993). Fat cell adrenergic receptors and the control of white and brown fat cell function 1 41. J. Lipid Res..

[58] Berlan M, Lafontan M (1985). Evidence that epinephrine acts preferentially as an antilipolytic agent in abdominal human subcutaneous fat cells: assessment by analysis of beta and alpha 2 adrenoceptor properties. Eur. J. Clin. Invest..

[59] Mahat B, Chassé É, Mauger JF, Imbeault P (2016). Effects of acute hypoxia on human adipose tissue lipoprotein lipase activity and lipolysis. J. Transl. Med..

[60] Michailidou Z (2015). Adipocyte pseudohypoxia suppresses lipolysis and facilitates benign adipose tissue expansion. Diabetes.

[61] de Glisezinski I (1999). Decrease of subcutaneous adipose tissue lipolysis after exposure to hypoxia during a simulated ascent of Mt everest. Pflügers Arch. Eur. J. Physiol..

[62] Nauck MA, Meier JJ (2016). The incretin effect in healthy individuals and those with type 2 diabetes: physiology, pathophysiology, and response to therapeutic interventions. Lancet Diabetes Endocrinol..

[63] Löfgren P (2002). Major gender differences in the lipolytic capacity of abdominal subcutaneous fat cells in obesity observed before and after long-term weight reduction. J. Clin. Endocrinol. Metab..

[64] Quintana-Gallego E (2004). Gender differences in obstructive sleep apnea syndrome: a clinical study of 1166 patients. Respir. Med..

[65] Gharib SA, Hayes AL, Rosen MJ, Patel SR (2013). A pathway-based analysis on the effects of obstructive sleep apnea in modulating visceral fat transcriptome. Sleep.

[66] Shaw JE (2016). The effect of treatment of obstructive sleep apnea on glycemic control in type 2 diabetes. Am. J. Respir. Crit. Care Med..

[67] Martinez-Ceron E (2016). Effect of continuous positive airway pressure on glycemic control in patients with obstructive sleep apnea and type 2 diabetes a randomized clinical trial. Am. J. Respir. Crit. Care Med..

[68] Loffler KA (2020). Continuous positive airway pressure treatment, glycemia, and diabetes risk in obstructive sleep apnea and comorbid cardiovascular disease. Diabetes Care.

[69] Authors/Task Force Members (2013). ESC guidelines on diabetes, pre-diabetes, and cardiovascular diseases developed in collaboration with the EASD. Eur. Heart J..

[70] Boston RC (2003). MINMOD millennium: a computer program to calculate glucose effectiveness and insulin sensitivity from the frequently sampled intravenous glucose tolerance test. Diabetes Technol. Ther..

