# Supplementary material for: Obstructive sleep apnoea increases lipolysis and deteriorates glucose homeostasis in patients with type 2 diabetes mellitus
Source: Sci Rep. 2021 Feb 11;11:3567. doi: 10.1038/s41598-021-83018-1 (PMC7878919; doi:10.1038/s41598-021-83018-1)
Supplement: Supplementary file 1 — Supplementary Information. [file 41598_2021_83018_MOESM1_ESM.pdf]

Obstructive sleep apnoea increases lipolysis and deteriorates glucose homeostasis in patients with type 2 diabetes mellitus

Minh Duc Trinh[1,2], Andrea Plihalova[3,4], Jan Gojda[3,4], Katerina Westlake[1], Jan Spicka[5], Zuzana Lattova[1], Martin Pretl[6] and Jan Polak[1,3]

[1] *Department of Pathophysiology, Third Faculty of Medicine, Charles University, Prague, Czech Republic.*
[2] *Department of Cardiology, University Hospital Královské Vinohrady, Prague, Czech Republic*
[3] *Department of Internal Medicine, University Hospital Královské Vinohrady, Prague, Czech Republic*
[4] *Centre for Research on Diabetes, Metabolism and Nutrition, Third Faculty of Medicine, Charles University, Prague, Czech Republic*
[5] *Department of Laboratory Diagnostics, University Hospital Královské Vinohrady, Prague, Czech Republic*
[6] *Neurology and Sleep Laboratory, INSPAMED s.r.o., Prague, Czech Republic*

Table A. IVGTT Results

	Non-adjusted data			Adjusted data for gender		
	**Control**	**T2DM**	**T2DM+OSA**	**Control**	**T2DM**	**T2DM+OSA**
Glucose (mmol/L)	5.54 (0.42)	7.68 (1.79) *	7.48 (1.58) *	5.49 (1.35)	7.67 (1.33) *	7.56 (1.39) *
HbA1c (mmol/mol)	36.21 (3.70)	52.70 (11.19) *	51.73 (12.47) *	36.00 (9.72)	52.63 (9.58) *	52.07 (9.86) *
Insulin (mU/L)	10.27 (4.00)	14.40 (4.30)	17.63 (8.90) *	10.71 (5.93)	14.57 (5.83)	16.84 (6.07) *
HOMA-IR	2.55 (1.06)	4.86 (1.80) *	5.94 (3.50) *	2.65 (2.29)	4.90 (2.25) *	5.75 (2.34) *
AIR_g ($mU.L^{-1}.min^{-1}$)	708.57 (458.12)	371.60 (590.20)	151.97 (170.02) *	745.23 (442.18)	385.60 (434.62)	86.64 (452.64) *
DI	1077.08 (642.85)	355.24 (576.91) *	174.16 (193.52) *	1099.12 (543.64)	363.66 (534.36) *	134.88 (556.50) *
S_I ($mU/L^{-1}.min^{-1}$)	1.69 (0.88)	1.58 (1.26)	1.21 (0.45)	1.70 (0.95)	1.59 (0.93)	1.18 (0.97)
S_G (min^{-1})	0.02 (0.01)	0.01 (0.01)	0.02 (0.01)	0.02 (0.01)	0.01 (0.01)	0.02 (0.01)
β-Cell function (mU/mM)	125.30 (45.31)	109.23 (90.55)	119.99 (81.48)	130.01 (72.64)	111.03 (71.40)	111.60 (74.36)
Insulin resistance ($mM.mU.L^{-2}$)	2.36 (0.95)	4.45 (1.42) *	5.47 (3.16) *	2.46 (2.00)	4.49 (1.97) *	5.28 (2.05) *

Data are mean (SD). HbA1c = glycated haemoglobin. HOMA-IR = homeostatic model assessment for insulin resistance. AIR_g – acute insulin response to glucose. DI – disposition index. S_I = insulin sensitivity. S_G = glucose effectiveness. * Significant difference ($p<0.05$) compared with control group.

Table B. IVGTT Results

	Non-adjusted data			Adjusted data for BMI		
	**Control**	**T2DM**	**T2DM+OSA**	**Control**	**T2DM**	**T2DM+OSA**
Glucose (mmol/L)	5.54 (0.42)	7.68 (1.79) *	7.48 (1.58) *	5.54 (1.34)	7.70 (1.35) *	7.47 (1.35) *
HbA1c (mmol/mol)	36.21 (3.70)	52.70 (11.19) *	51.73 (12.47) *	36.21 (9.60)	52.62 (9.71) *	51.81 (9.74) *
Insulin (mU/L)	10.27 (4.00)	14.40 (4.30)	17.63 (8.90) *	10.34 (5.32)	15.14 (5.38) *	16.79 (5.39) *
HOMA-IR	2.55 (1.06)	4.86 (1.80) *	5.94 (3.50) *	2.57 (2.11)	5.07 (2.14) *	5.96 (2.15) *
AIR_g ($mU.L^{-1}.min^{-1}$)	708.57 (458.12)	371.60 (590.20)	151.97 (170.02) *	710.61 (445.44)	391.39 (450.39)	129.33 (451.92) *
DI	1077.08 (642.85)	355.24 (576.91) *	174.16 (193.52) *	1078.75 (535.16)	371.43 (541.10) *	155.65 (542.95) *
S_I ($mU/L^{-1}.min^{-1}$)	1.69 (0.88)	1.58 (1.26)	1.21 (0.45)	1.68 (0.89)	1.51 (0.90)	1.30 (0.91)
S_G (min^{-1})	0.02 (0.01)	0.01 (0.01)	0.02 (0.01)	0.02 (0.01)	0.01 (0.01)	0.02 (0.01)
β-Cell function (mU/mM)	125.30 (45.31)	109.23 (90.55)	119.99 (81.48)	126.10 (66.70)	117.02 (67.44)	111.08 (67.67)
Insulin resistance ($mM.mU.L^{-2}$)	2.36 (0.95)	4.45 (1.42) *	5.47 (3.16) *	2.38 (1.89)	4.62 (1.91) *	5.27 (1.92) *

Data are mean (SD). HbA1c = glycated haemoglobin. HOMA-IR = homeostatic model assessment for insulin resistance. AIR_g – acute insulin response to glucose. DI – disposition index. S_I = insulin sensitivity. S_G = glucose effectiveness. * Significant difference ($p<0.05$) compared with control group.

Table C. IVGTT Results

	Non-adjusted data			Adjusted data for gender, BMI and waist circumference		
	**Control**	**T2DM**	**T2DM+OSA**	**Control**	**T2DM**	**T2DM+OSA**
Glucose (mmol/L)	5.54 (0.42)	7.68 (1.79) *	7.48 (1.58) *	5.31 (1.40)	7.70 (1.33) *	7.78 (1.46) *
HbA1c (mmol/mol)	36.21 (3.70)	52.70 (11.19) *	51.73 (12.47) *	35.61 (10.57)	52.56 (9.99) *	52.63 (10.94) *
Insulin (mU/L)	10.27 (4.00)	14.40 (4.30)	17.63 (8.90) *	11.29 (5.49)	15.27 (5.21)	15.33 (5.73)
HOMA-IR	2.55 (1.06)	4.86 (1.80) *	5.94 (3.50) *	2.69 (2.30)	5.11 (2.19) *	5.48 (2.40) *
AIR_g ($mU.L^{-1}.min^{-1}$)	708.57 (458.12)	371.60 (590.20)	151.97 (170.02) *	773.70 (467.74)	403.35 (443.90)	29.03 (487.78) *
DI	1077.08 (642.85)	355.24 (576.91) *	174.16 (193.52) *	1076.97 (582.70)	383.12 (553.00) *	146.44 (607.65) *
S_I ($mU/L^{-1}.min^{-1}$)	1.69 (0.88)	1.58 (1.26)	1.21 (0.45)	1.55 (0.92)	1.53 (0.88)	1.45 (0.96)
S_G (min^{-1})	0.02 (0.01)	0.01 (0.01)	0.02 (0.01)	0.02 (0.01)	0.01 (0.01)	0.01 (0.01)
β-Cell function (mU/mM)	125.30 (45.31)	109.23 (90.55)	119.99 (81.48)	140.01 (68.08)	117.97 (64.61)	90.65 (71.00)
Insulin resistance ($mM.mU.L^{-2}$)	2.36 (0.95)	4.45 (1.42) *	5.47 (3.16) *	2.52 (2.05)	4.65 (1.94) *	5.03 (2.13) *

Data are mean (SD). HbA1c = glycated haemoglobin. HOMA-IR = homeostatic model assessment for insulin resistance. AIR_g – acute insulin response to glucose. DI – disposition index. S_I = insulin sensitivity. S_G = glucose effectiveness. * Significant difference ($p<0.05$) compared with control group.

Table D. Gender differences in adipose tissue lipolysis

	Control		T2DM		T2DM+OSA	
	**Male**	**Female**	**Male**	**Female**	**Male**	**Female**
Spontaneous lipolysis (μmol/l)	55.98 (3.93)	50.61 (10.66)	40.29 (16.70)	43.34 (7.85)	58.14 (31.16)	60.95 (11.73)
Epinephrine-stimulated lipolysis (μmol/l)	152.13 (8.76)	122.51 (41.56)	168.55 (25.47)	166.31 (62.44)	277.23 (98.49)	169.50 (75.36)
Isoprenaline-stimulated lipolysis (μmol/l)	148.37 (27.72)	164.89 (34.99)	213.75 (86.95)	180.01 (42.00)	184.38 (25.06)	155.88 (62.02)

Data are mean (SD). Data present dialysate glycerol concentration. There is no significant difference between genders among groups, $p>0.05$ for all comparisons.
